# Sensation Seeking and Gambling Behavior in Adolescence: Can Externalizing Problems Moderate this Relationship?

**DOI:** 10.3390/ijerph17238986

**Published:** 2020-12-02

**Authors:** Franca Tani, Lucia Ponti, Simon Ghinassi

**Affiliations:** 1 Department of Health Sciences, University of Florence, 50139 Florence, Italy; franca.tani@unifi.it; 2 Department of Education, Languages, Intercultures, Literatures and Psychology, University of Florence, 50121 Florence, Italy; simon.ghinassi@outlook.it

**Keywords:** gambling behaviors, adolescence, sensation seeking, externalizing problems

## Abstract

Gambling is a widespread phenomenon during adolescence. Among different risk factors involved in the onset of adolescent gambling behaviors, one factor that is studied is the sensation seeking personality trait. However, the literature is heterogeneous and a direct relationship between sensation seeking and gaming behaviors has not always been highlighted. This suggests that the relationship can be influenced by other factors. In particular, we explored the moderating role of externalizing problems in this relationship. A total of 363 adolescents (232 males and 131 females) aged 14 to 20 (M = 16.35, SD = 1.36) completed a battery of questionnaires aimed to assess their gambling behaviors, as well as the levels of externalizing problems and sensation seeking. The results showed that sensation seeking was associated with gambling severity, but this relationship was significant when externalizing problems were high and medium. On the contrary, when externalizing problems were low, the relationship between sensation seeking and gambling severity was not significant. Overall, sensation seeking in adolescence can favor the implementation of risk behaviors, such as gambling, but only in association with the presence of externalizing problems. Limitations, strengths, and social and clinical implications of the present study are discussed.

## 1. Introduction

Adolescence is a crucial transitional period characterized by significant physical, cognitive, emotional, and social changes [1]. In particular, it represents a vulnerable phase to maladjustment [2] and the implementation of addiction and risk-taking behaviors [3,4]. This is due to the fact that, in general, adolescents lack recognition and/or awareness of the potential negative effects of risk behavior, have a self-perceived invulnerability, and have a high level of sensation seeking and risk-taking, to which a not fully developed cognitive ability may also be added [3,5,6,7]. Therefore, it is not surprising that, as often happens with other forms of addiction [4], adolescence is a critical period in which gambling behaviors arise and can become problematic [8]. Gambling behaviors themselves represent a risk-taking activity because they include those behaviors that involve betting money or items of material value on an event with an uncertain outcome in the hope of obtaining further money and/or material goods [9]. In this regard, recent literature has highlighted that gambling is spreading widely among adolescents, so much so that it has become a serious social and public health problem [10,11,12,13] with deleterious psychological, social, relational, and financial consequences, both in the short- and long-term [3,14,15]. In particular, a recent systematic review conducted by Calado et al. [11] showed that 0.2–12.3% of adolescents meet the criteria for being classified as problem gamblers. In the Italian context, the country of the present study, the percentage of adolescents who gamble has increased rapidly in recent years. For example, Chiesi et al. [16] showed that 17% and 7% were classified as at-risk or problematic gamblers, respectively. A successive study highlighted higher percentages, showing that, although 73.2% of Italian adolescents do not have gambling problems, 18.5% can be classified as at-risk gamblers and 8.3% as problematic gamblers [17]. More recently, Cosenza, Ciccarelli, and Nigro [18] found even higher percentages of gamblers among Italian adolescents, showing that 20.2% and 9.8% can be defined as at-risk or problematic gamblers, respectively.

These data are alarming given that, in many countries, including Italy, the law prohibits minors from engaging in gambling activities. This rapid increase may be due to the fact that, in addition to the very large and widespread dissemination of slot machines and scratch card dealers, in recent years, technological and information technology developments have favored the proliferation of a large number of games and apps linked to gambling. These games and apps are easily accessible and available even to minors since access restrictions are easily circumvented [3].

Given the high prevalence of gambling among adolescents and the negative consequences it can have, it is not surprising that the literature has focused on the recognition of risk factors involved in the development of gambling problems in adolescents [19,20,21,22]. One of the most studied risk factors involved in the onset of gambling is sensation seeking, which is the tendency to pursue “varied, novel, complex, and intense sensations and experiences, and the willingness to take physical, social, legal, and financial risks for the sake of such experiences” [23] (p. 27). In this regard, many studies have found a relationship between sensation seeking and gambling severity in adolescents. For example, a study conducted by Donati et al. [24] highlighted that sensation seeking was a significant predictor of at-risk and problematic gambling. Similarly, Estevez et al. [25] found that sensation seeking was high in young gamblers, as did Reardon et al. [26], who highlighted a positive correlation between these two variables. Another recent study found that one of the antecedents of regular gambling was high sensation seeking scores [27]. Moreover, Donati et al. [28] showed that sensation seeking has a significant direct effect on gambling severity, with higher levels of sensation seeking being predictors of greater severity. Briefly, such evidence suggests that the pursuit of novelty and intense stimulation, typical of individuals with high scores in sensation seeking, may explain why some youth are attracted to gambling. In fact, gambling often represents an escape from everyday life, guided by curiosity for new experiences and desire for excitement deriving from the unpredictability of those experiences [29,30].

However, not all studies in the literature agree on this aspect, and some have found no direct role of sensation seeking in gambling severity [31,32]. Considering these heterogeneous data, one may surmise that there are additional variables that could moderate this relationship. To this point, Calderia et al. [33] found that the direct role of sensation seeking in gambling was completely attenuated when considering its indirect path through the frequency of alcohol and drug use. In our opinion, however, the presence of general externalizing problems, as well as alcohol and drug use, can intervene in the relationship between sensation seeking and gambling. Externalizing problems, including the violation of age-appropriate rules and expectations, interpersonal conflict, oppositionality, aggression, and impulsiveness, can be considered a construct that includes both aggressive and delinquent behaviors [34]. The relevant literature has shown that externalizing problems increases in prevalence during adolescence and can be considered a predictor of antisocial behavior in adults [35,36]. Furthermore, a plethora of studies has highlighted that these types of problems are connected to both sensation seeking and gambling severity. In particular, sensation seeking has been found to be a predictor of a wide range of externalizing problem behaviors in adolescence [23,37] and is strongly related with antisocial and delinquent behavior [38,39,40]. 

Regarding gambling, a meta-analysis of longitudinal studies has pointed out that antisocial behaviors, violence, and uncontrolled temperament represent some early risk factors for the development of gambling problems [20]. In addition, several studies have shown that gambling severity was significantly associated with marked externalizing problems [10,41], and Allami et al. [19] verified that adolescents showing externalizing profiles at 12 years of age reported a large number of gambling-related problems at 16 and 23 years old. 

**Figure 1:**
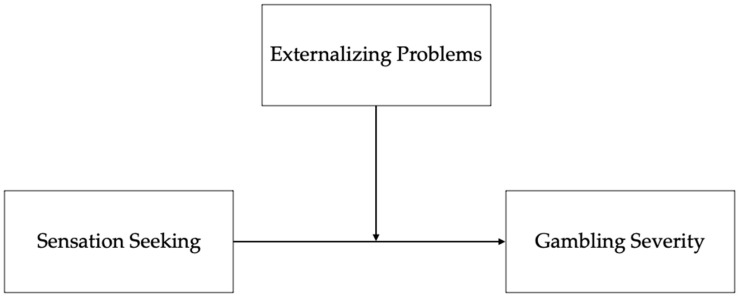
Conceptual model tested.

Starting from these considerations, the main aim of the present study was to explore the moderating role of externalizing problems in the relationship between sensation seeking and gambling severity. Our conceptual model is reported in Figure 1.

In particular, we hypothesized that the level of sensation seeking was significantly linked to the severity of gambling behaviors. However, we supposed that this relationship was greater in the presence of externalizing problems, and that it could be insignificant in the absence of externalizing problems. 

## 2. Materials and Methods

### 2.1. Participants

A total of 363 adolescents (232 males and 131 females) between the ages of 14 to 20 (M = 16.35, SD = 1.36), who were attending two high schools in the metropolitan area of Florence, were recruited for the present study. More than 93% of the students came from central Italy from families characterized by a middle/high socio-educational background, with more than 59% of fathers and 72% of mothers having a high school diploma or university degree. In addition, 95% of fathers and more than 75% of mothers had a job.

### 2.2. Procedure

A cross-sectional study was conducted in accordance with the guidelines for the ethical treatment of human participants of the Italian Psychological Association. First, the Ethical Committee of the University of Florence approved the study (n. 81120/2018). Second, written authorization was obtained by the principals of the two high schools, selected according to a casual criteria applied to all high schools in the metropolitan area of Florence. Then, all students were informed of the aims of the study, that participation was anonymous, that they could withdraw at any time, and that their participation was voluntary without any reward. All students signed informed consent and, in case of minor students, informed consent was signed by parents. Data collection was performed in class during normal school hours. Specifically, two trained researchers went to the classrooms and collectively administered the questionnaires in paper form to the students.

### 2.3. Measures

The Italian version of the South Oaks Gambling Screen Revised for Adolescents (SOGS-RA) [16], developed by Winters et al. [42], was used to assess gambling behavior. The SOGS-RA is a self-report instrument composed of two parts. The first part measures the frequency of gambling, the typology of gambling activity, and the amount of money spent on gambling the previous year. From the first part, we obtained the percentage of adolescents who reported having gambled during the previous year. The second part of the instrument is composed of 12 dichotomous items. The total score, obtained by the sum of these items, measures the severity of gaming behaviors as a continuous variable. The Cronbach’s alpha for the present sample was 0.71, which represents an acceptable value [43].

The Italian version of the Youth Self Report (YRS) [34,44] was employed to assess the level of externalizing behavior problems. The externalizing scale of the YRS is composed of two syndrome scales (delinquent and aggressive behavior scales) consisting of 30 items rated on a three-point scale, from zero (not true) to two (very true or often true). For the present sample, the Cronbach’s alpha was 0.84 for the externalizing scale, which represents a good value [43].

The Italian version of the Brief Sensation Seeking Scale (BSSS) [45], developed by Zuckerman et al. [46], was used to measure sensation seeking traits. The BSSS is a self-report instrument composed of eight items rated on a five-point Likert scale, from one (totally disagree) to five (totally agree). For the present study, the Cronbach’s alpha was 0.71, which represents an acceptable value [43].

### 2.4. Data Analysis

The collected data were analyzed using SPSS version 23.0 (IBM, Armonk, NY, USA). Missing data were completely random (Little’s Missing Completely At Random (MCAR) test: χ^2^ = 80.51, df = 98; *p* = 0.900) and an expectation maximization (EM) algorithm was employed to substitute missing items. Descriptive statistics and pairwise correlation coefficients were performed. The normality of each variable was explored using Curran and colleagues’ criteria that stabilized an accepted range for skewness of ±2 and kurtosis of ±7 [47]. Regarding Pearson’s correlations, Cohen’s criteria were used [48], which indicate correlations around 0.10, near 0.30, and 0.50 or higher, which are small, medium, and large effect sizes, respectively. Then, prior to addressing the aims of the study, gender effect on gambling behaviors, externalizing behavior problems, and sensation seeking were controlled by performing a series of t-tests, and the corresponding effect sizes were reported (Cohen’s d). Finally, in order to explore the role of externalizing problems on the relationship between sensation seeking and gambling severity, a hierarchical regression analysis consisting of four consecutive steps was carried out, with gambling as the dependent variable. These analyses were performed following Aiken and West’s procedure [49]. Sensation seeking and externalizing problems were centered at the sample mean for both main effect and interaction terms to reduce potential multi-collinearity. Then, for the first hierarchical regression, gender as a dummy variable was entered in Step 1, sensation seeking was entered in Step 2, externalizing problems (moderator variables) were entered in Step 3, and the two-way interaction between sensation seeking and externalizing problems was entered in Step 4.

Significant interaction between independent variables (sensation seeking) and moderating variables (externalizing problems) was graphically represented using ModGraph [50], and moderating variables and independent variables were represented as low (values 1 SD below the mean), medium (values ranging between 1 SD below mean and 1 SD above mean), or high (values 1 SD above the mean). Finally, simple slope analyses were conducted using post-hoc regression to explore the significance of each slope. In this regard, the externalizing problem variable was standardized and three groups were considered: low level of externalizing problems (values < −1); medium level of externalizing problems (values ranging from −1 to 1); and high level of externalizing problems (values > 1).

## 3. Results

Regarding gambling, data collected by the first part of the SOGS-RA showed that more than 67% of adolescents (*n* = 245) declared that they had gambled at least once in the previous 12 months. In particular, 193 (78.8%) were minors. Table 1 shows the descriptive statistics and pairwise correlation coefficients of externalizing problems, sensation seeking, and gambling, as collected using the second part of the SOGS-RA as continuous variables. Therefore, in subsequent analyses, all participants were included.

All variables presented a normal distribution. Moreover, higher levels of externalizing problems were linked to higher levels of sensation seeking and gambling severity. Finally, the level of sensation seeking presented a medium and positive correlation with the level of gambling problems.

Significant differences emerged between males and females on all variables. In particular, males reported higher levels of externalizing problems (males: M = 11.78, SD = 7.10; females: M = 9.37, SD = 5.82; t (361) = 3.30, *p* = 0.000, Cohen’s d = 0.37), sensation seeking (males: M = 26.59, SD = 5.92; females: M = 23.95, SD = 5.34; t (361) = 4.23, *p* = 0.000, Cohen’s d = 0.47), and gambling severity (males: M = 1.23, SD = 1.70; females: M = 0.09, SD = 0.31; t (361) = 7.57, *p* = 0.000, Cohen’s d = 0.93). Given these gender differences, gender was inserted as a control variable in the subsequent analyses.

Table 2 shows the results of the first hierarchical regression in reference to the moderating role of externalizing problems in the relationship between sensation seeking and gambling severity.

**Figure 2:**
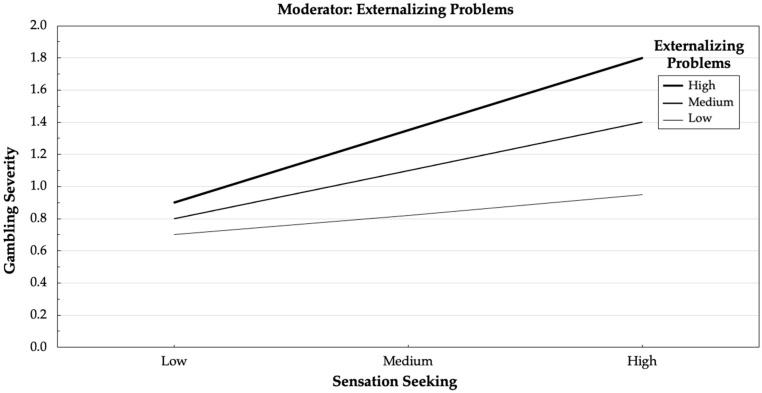
Interaction between sensation seeking and externalizing problems in the prediction of gambling severity.

In Step 1, gender accounted for 14% of the variance in gambling severity, F (1, 361) = 57.29, *p* = 0.000. In Step 2, sensation seeking explained 0.04% of additional variance, F (2, 360) = 37.82, *p* = 0.000. In Step 3, the moderating variable of the level of externalizing problems explained 0.03% of additional variance, F (3, 359) = 29.73, *p* = 0.000. Finally, in Step 4, the interaction terms explained 0.02% of additional variance, F (4, 358) = 24.53, *p* = 0.000. Higher levels of sensation seeking were more strongly associated with higher levels of gambling severity at higher levels of externalizing problems. This interaction is shown in Figure 2.

Post hoc analyses showed that the relationship between sensation seeking and gambling severity was significant when externalizing problems were medium (β = 0.26, *p* = 0.000) and high (β = 0.35, *p* = 0.002). On the contrary, the relationships were non-significant when externalizing problems were low (β = 0.12, *p* = 0.323).

## 4. Discussion

The main focus of the present study was to explore the relationship between sensation seeking traits and gambling severity in adolescents by exploring the moderating role played by the presence of externalizing problems in this relationship. We hypothesized that the seeking of novel and intense stimulation could represent a significant risk factor to the development of gambling problems. However, considering the results of previous studies, we also posited that the levels of externalizing problems manifested by adolescents could play a significant role as a moderating variable in this relationship. Specifically, we assumed that this relationship may be significantly greater in the presence of externalizing problems and may not be significant in their absence. In other words, since externalizing problems are linked to impulsive and uncontrolled behaviors, they can represent a risk factor in the relationship between the sensation seeking trait and gambling severity.

In line with recent literature, our results showed a relevant and alarming picture, highlighting that gambling is a very common phenomenon among adolescents [13,16,17,18,51]. In fact, more than 67% of the adolescents in our sample declared that they had gambled at least once in the previous year. Moreover, of the adolescent gamblers, 78.8% (*n* = 193) were minors, despite the fact that, according to current legislation, it is illegal for them to gamble. These results are in line with previous studies conducted in the Italian context on gambling among adolescent minors and those of legal age [13,16].

Furthermore, results highlight significant gender differences, confirming that externalizing behaviors constitute a problematic aspect especially for males. In fact, in accordance with the existing literature, we found that males reported higher levels of sensation seeking [52,53,54], of externalizing problems [55,56,57], and of gambling [3,10,21,58,59,60] than females.

In addition, our results highlight that gambling severity are significantly and positively correlated both with high levels of sensation seeking and with the presence of externalized problems. These data are in line with studies present in the literature that highlight that the desire for excitement, novelty, and intense stimulation, characterizing individuals who have high levels of sensation seeking, is closely connected to gambling severity [24,25,26,27,28]. In addition, the presence of aggressive and delinquent behaviors, typical of an externalizing profile, are also correlated to gambling severity in adolescence [10,19,20,41].

Finally, although the presence of sensation seeking was related to the severity of gambling behaviors, our results show that this relationship was moderated by the levels of externalizing problems experienced by adolescents. In particular, the relationship between sensation seeking and gambling severity was found to be significant only when adolescents show medium or high externalizing problems while, on the contrary, this relationship disappears when the externalizing problems are low. These results point out the risk of sensation seeking in the development of antisocial and uncontrolled behaviors in adolescents. However, it could be that sensation seeking alone is not a significant risk factor in gambling activity when the adolescent does not present externalizing problems.

These results provide a significant contribution to the knowledge of risk factors in adolescent gambling; however, the present study presents some limitations. The first limitation is related to the convenience sample used in the study. It would be useful for future research to extend recruitment to a more heterogeneous sample (e.g., provenience and socio-educational background) since the present results are based on a sample of adolescents mostly from central Italy and from families characterized by a middle/high socio-educational background. The second limitation concerns the nature of the data, given that they are based only on self-report questionnaires that may not fully reflect true adolescent gambling behavior due to possible biases deriving from the social stigma associated with such behavior. The third limitation is that measures of social desirability have not been considered, and this could be a problem, as the participants could have difficulty admitting their gambling problems and/or the presence of an aggressive or delinquent nature. Moreover, since this is a cross-sectional study, it is impossible to determine the direction of the observed effects and to infer casual relations. Another limitation is the use of the SOGS-RA to assess the presence and severity of gambling behaviors, considering issues of item content and false positives [61]. However, in addition to the fact that the evidence from item response theory supports the reliability and suitability of the SOGS-RA as a screening tool in adolescents [16], we used the questionnaire results only as continuous measures in the data analysis. Another limitation is linked to the amount of variance explained. Although the results were significant, the variance explained is very small. This may be due to the fact that the proposed theoretical model is not exhaustive, and other variables may certainly play a significant role in the interaction between sensation seeking and gambling. For example, the attachment relationship with parents appears to be an important factor in the onset of problematic gambling behavior [62].

Despite these limitations, our results have relevant social and clinical implications. From a social point of view, we found that most adolescents, even minors, gamble. From a social policy point of view, it would be helpful to have greater legal control of gambling operations and to implement gambling prevention programs among adolescents.

Moreover, from a clinical point of view, our results underline the importance for clinicians who deal with adolescents with gambling problems to pay attention to the personality traits that characterize them. Above all, clinicians should work on aspects related to individual well-being, in order to promote the reduction of problems underlying deviant and antisocial behaviors. In line with this goal, it would be useful for future research to continue to investigate other variables involved in adolescent gambling problems. In fact, this would be very useful information for clinicians to better direct their work and more effectively help adolescents who are involved in gambling behaviors that could become more problematic with increasing age. Gambling in adolescence is considered a risk factor for a wide range of negative consequences, both in the short- and long-term [14], and it is often associated with gambling problems in adulthood [63,64,65]. Therefore, this research area should be further studied in future investigations.

## 5. Conclusions

In the present study we explored the moderating role of externalizing problems in the relationship between sensation seeking and gambling severity using a representative sample of Italian adolescents. Overall, results highlighted that the level of sensation seeking trait in adolescence was significantly associated to gambling severity. However, this relationship was significant only in presence of high level of externalizing problems. In other words, externalizing problems represent a significant risk factor for the severity of gambling behaviors in adolescence. In fact, without the presence of these problems, the relationship between sensation seeking and gambling was insignificant. 

## Figures and Tables

**Table 1:** Descriptive statistics and pairwise correlation coefficients for all variables.

	M	SD	Skewness	Kurtosis	1	2	3
1. Externalizing problems	10.91	6.75	1.00	1.17	-	0.44 **	0.29 **
2. Sensation seeking	25.64	5.84	−0.09	−0.30		-	0.27 **
3. Gambling	0.82	1.48	2.05	3.74			-

** *p* < 0.01.

**Table 2:** Hierarchical regression analysis results for externalizing problems and sensation seeking as predictors of gambling severity.

	ß	t	*p*	95% CI	
Step 1					
Gender	−0.37	−7.58	0.000	−1.43	−0.84
Step 2					
Gender	−0.33	−6.67	0.024	−1.30	−0.71
Sensation seeking	0.20	4.00	0.000	0.03	0.07
Step 3					
Gender	−0.31	−6.45	0.000	−1.26	−0.67
Sensation seeking	0.12	2.26	0.024	0.01	0.06
Externalizing problems	0.18	3.37	0.001	0.02	0.06
Step 4					
Gender	−0.31	−0.46	0.000	−1.25	−0.66
Sensation seeking	−0.08	−0.88	0.382	−0.07	0.03
Externalizing problems	0.13	2.43	0.016	0.01	0.05
Sensation seeking X Externalizing problems	0.26	2.72	0.007	0.00	0.01
